# Fluorescence *in situ* hybridisation analysis of chromosomal aberrations in gastric tissue: the potential involvement of *Helicobacter pylori*

**DOI:** 10.1038/sj.bjc.6602533

**Published:** 2005-04-12

**Authors:** L Williams, G J S Jenkins, S H Doak, P Fowler, E M Parry, T H Brown, A P Griffiths, J G Williams, J M Parry

**Affiliations:** 1 Neath Port Talbot Hospital, Baglan, UK; 2 Swansea Clinical School, University of Wales, Swansea, UK; 3 School of Biological Sciences, University of Wales, Swansea, UK; 4 Department of Surgery, Morriston Hospital, Swansea, UK; 5 Department of Pathology, Morriston Hospital, Swansea, UK

**Keywords:** gastric cancer, *Helicobacter pylori*, aneuploidy, reactive oxygen species

## Abstract

In this series of experiments, a novel protocol was developed whereby gastric cells were collected using endoscopic cytology brush techniques, and prepared, such that interphase fluorescence *in situ* hybridization (FISH) could be performed. In total, 80 distinct histological samples from 37 patients were studied using four chromosome probes (over 32 000 cells analysed). Studies have previously identified abnormalities of these four chromosomes in upper GI tumours. Using premalignant tissues, we aimed to determine how early in Correa's pathway to gastric cancer these chromosome abnormalities occurred. Aneuploidy of chromosomes 4, 8, 20 and 17(p53) was detected in histologically normal gastric mucosa, as well as in gastritis, intestinal metaplasia, dysplasia and cancer samples. The levels of aneuploidy increased as disease severity increased. Amplification of chromosome 4 and chromosome 20, and deletion of chromosome 17(p53) were the more common findings. Hence, a role for these abnormalities may exist in the initiation of, and the progression to, gastric cancer. *Helicobactor pylori* infection was determined in premalignant tissue using histological analysis and PCR technology. Detection rates were comparable. PCR was used to subtype *H. pylori* for CagA status. The amplification of chromosome 4 in gastric tissue was significantly more prevalent in *H. pylori*-positive patients (*n*=7) compared to *H. pylori*-negative patients (*n*=11), possibly reflecting a role for chromosome 4 amplification in *H. pylori*-induced gastric cancer. The more virulent CagA strain of *H. pylori* was associated with increased disease pathology and chromosomal abnormalities, although numbers were small (CagA+ *n*=3, CagA− *n*=4). Finally, *in vitro* work demonstrated that the aneuploidy induced in a human cell line after exposure to the reactive oxygen species (ROS) hydrogen peroxide was similar to that already shown in the gastric cancer pathway, and may further strengthen the hypothesis that *H. pylori* causes gastric cancer progression via an ROS-mediated mechanism.

Gastric cancer is the second most deadly cancer worldwide, accounting for 649 000 deaths each year and 876 000 incident cases in the world each year (Parkin *et al*, 2001). In the UK, it is the fifth most frequent cancer in men and the ninth in women (www.cancerresearchuk.org/cance rstats/).

Gastric cancer is often diagnosed at an advanced stage when symptoms such as weight loss, abdominal pain, vomiting and gastrointestinal bleeding finally alert patients to seek medical advice. Treatment regimens, such as surgery, chemotherapy and radiotherapy, are therefore limited by the extent of advanced local disease and metastatic spread. In fact, only 55–65% of gastric cancers are surgically resectable at diagnosis (Keighley, 2003). Hence, the prognosis of gastric cancer is poor, helping to make gastric cancer a leading cause of cancer death worldwide (Parkin *et al*, 2001). The early detection of gastric cancer (or a premalignant form with a high risk of progression) could improve the current poor survival rates. Correa (1988) has outlined a multistep pathway to gastric adenocarcinoma, involving precancerous stages, gastritis and intestinal metaplasia, and hence, it may be that finding molecular markers, which are associated with these early histological changes, could improve prognosis.

It has been well established that chromosome abnormalities are implicated in the initiation and progression of many forms of cancer, including gastric cancer (Lengauer *et al*, 1997; Nardone *et al*, 1999). Aneuploidy (abnormal chromosome numbers) of varying degrees has previously been documented in all stages of gastric cancer (Beuzen *et al*, 2000). In an effort to assess whether early chromosome abnormalities might represent suitable diagnostic molecular markers of gastric cancer, we undertook a cytogenetic evaluation of all histological stages of gastric cancer. Specifically, we used previously published cytogenetic data from upper GI tract cancers produced by us, and others, to see how early in premalignant gastric cancer, these events occurred and if they could be linked to infection with *Helicobactor pylori*. After an extensive literature search for chromosome abnormalities in upper GI tract cancer, we chose four of the most common or most interesting chromosomes for study (we limited the study to four chromosomes due to time constraints). We chose here to study copy number abnormalities of chromosomes 4, 8, 20 and the p53 locus (chromosome 17) over the full histological range of gastric cancer. Aneuploidy involving chromosome 20 and 8 has been shown in several studies of gastric cancer tissue (Kokkola *et al*, 1998; Beuzen *et al*, 2000; Kitayama *et al*, 2000; Koo *et al*, 2000; Okada *et al*, 2000; Wu *et al*, 2001). Chromosome 4 aneuploidy has also been implicated in another upper GI malignancy, oesophageal, by our group (Croft *et al*, 2002; Doak *et al*, 2003). P53 is the most frequently mutated gene in human cancer (Levine *et al*, 1991; Tamura *et al*, 1991; Vogelstein and Kinzer, 1992; Sipponen *et al*, 1998) and is commonly found to be abnormal in 30–58% of intestinal and diffuse gastric tumours (Grady, 2001). Hence, here we assessed whether copy number changes of these four chromosomes occurred in gastritis or intestinal metaplasia, as well as dysplasia and adenocarcinoma.

A number of agents are thought to play a role in the aetiology of gastric cancer and include the infectious agent *H. pylori.* In 1994, the International Agency for Research on Cancer (IARC) declared *H. pylori* a class one human carcinogen, capable of inducing changes leading to gastric cancer (IARC, 1994). *Helicobator pylori* is linked causally to gastric cancer and we know that the production of reactive oxygen species (ROS) is one way that the bacterium exerts its effect on gastric cells (Correa, 1988; Asaka *et al*, 1997; Chan *et al*, 1999; Obst *et al*, 2000). Indeed, O’Connor and Sebastian (2003) suggested that *H. pylori* infection may account for 60% of all gastric cancers worldwide. There is now a large body of evidence linking *H. pylori* to gastric cancer with epidemiological studies showing a 3–12-fold increased risk of gastric cancer in those people infected with *H. pylori* (Cover and Blaser, 1995). *Helicobactor pylori* strains with the CagA pathogenicity island are known to be more virulent, producing more severe pathological infection in humans (Blaser, 1998). CagA+ strains are highly immunogenic strains of *H. pylori* and are associated with increased cytokine expression (Crabtree *et al*, 1995), inflammatory cell infiltrate and release of ROS adjacent to gastric mucosa (Fiocca *et al*, 1994; Sipponen *et al*, 1998). The oxidative DNA damage induced by ROS is an important mechanism in carcinogenesis. It has been implicated in up to 50% of all human cancers (Beckman and Ames, 1997) and importantly, has been considered a potential causative agent in gastric cancer (Correa and Shiao, 1994; Stadtlander and Waterbor, 1999). Therefore, we also examined here the effect of ROS exposure on chromosome stability *in vitro*. We exposed human cells to subtoxic doses of hydrogen peroxide (H_2_O_2_), and used the micronucleus assay and fluorescence *in situ* hybridization FISH to assess chromosome damage. The micronucleus assay is routinely used to study chromosome damage (Fenech *et al*, 1999) and can detect chromosome fragmentation (induction of micronuclei) as well as whole chromosome loss (using kinetochore staining).

## METHODS

### Patient enrolment into the study

Patients attending Neath Port Talbot Hospital for upper GI endoscopy were invited to participate in this study. The hospital is situated in a mixed rural and urban area of West Wales, and serves a socially deprived population of approximately 140 000. Ethical Approval was obtained from the local ethics committee and samples were only taken from consenting adults. Exclusions to the study were made when consent could not be adequately given, when the patient was taking proton pump inhibitors or nonsteroidal anti-inflammatory drugs, had undertaken recent *H. pylori* eradication or had a history of previous upper GI surgery. Information was collected on sex, age, ethnicity, family history, diet, smoking, alcohol and drug intake, prior to the endoscopy.

### Endoscopic cytology brushings

Cytology brushes (Diagmed Ltd., Thirsk, UK) were used to collect cells from the gastric and oesophageal mucosa. This methodology has previously been described by us, for use in analysing oesophageal samples (Doak *et al*, 2003). The advantages of using cytology brushes to collect cells for cytogenetic studies include the broader area from which the cells are collected and the fact that primarily only epithelial cells are sampled. Gastric brushes were taken at the same site as the gastric biopsies used for histological diagnosis and PCR detection of *H. pylori*. Gastric brushes were targeted at macroscopically abnormal stomach when present, otherwise antral brushes were taken. Oesophageal brushes were taken from macroscopically normal distal oesophagus for comparison. The area of stomach to be sampled was washed with 20 ml sterile water prior to brushing. Brushes were also taken prior to biopsy, to minimise bleeding. No patient reported any immediate complications following this procedure.

Optimisation of the collection of gastric cells for interphase FISH was extensively carried out, the optimal protocol is described here. Brushes were stored in 10 ml of 90% methanol, on ice, in the Endoscopy Unit prior to transportation to the laboratory. Vigorous shaking of the brushes to dislodge the cells from the bristles immediately after storage in methanol was found to improve cell yield. These brushes were stored successfully for up to a week.

### Cytology brushes from surgical resections of gastric cancer

Surgical resection specimens from 10 patients undergoing gastrectomy for gastric cancer at Morriston Hospital, Swansea were identified by a Consultant Pathologist (APG). Multiple brushings from the surgical resection specimens were taken, together with biopsies at the same sites, hence allowing histological diagnosis. The cytology brushes were stored in 90% methanol within 30 min of resection.

### Interphase cell preparations

The cell suspensions were washed three times by centrifugation/resuspension in ETN buffer (0.1 M EDTA, 10 mM Tris-HCl pH7, 20 mM NaCl) (Doak *et al*, 2003). The resultant cell pellet was resuspended in 0.5 ml ETN buffer and a cytodot was produced on a glass slide using a Cytospin 4 (ThermoShandon, Cheshire, UK). Larger pellets were resuspended in 1 ml ETN buffer. The cytodot was examined with a light microscope, and cell suspensions were diluted accordingly to ensure that an adequate number of cells, evenly spaced apart, were present for interphase FISH. Oesophageal cells were easily visualised, but the gastric cells frequently had no clear cytoplasm/cell membrane, appearing to be partially digested. This improved when the brushes were stored in 90% methanol, and not ETN, giving a better yield of cells. Cell preparations on slides were fixed in 90% methanol at −20°C for 10 min and left to air dry. An average of 2–5 slides were produced per sample. Interphase cell preparations were incubated at 37°C for 10 min and then treated with 300 *μ*l ml^−1^ HCl pepsin, pH 2.7–3 (Sigma, Dorset, UK) at 37°C, to remove cytoplasmic proteins, hence improving probe penetration. Oesophageal cells were treated for 7 min as described by Doak *et al*, 2003, but over this period of time overdigestion of gastric cells resulted in loss of nuclei. Again, this suggested that partial gastric cell digestion had already occurred, therefore the pepsin digestion time was reduced to 5 min and the yield improved. Slides were washed in PBS (5 min) and PBS/MgCl_2_ (5 min) at room temperature to arrest the enzymatic action of pepsin. They were then dehydrated in increasing concentrations of ethanol prior to FISH.

### Interphase FISH

Centromeric enumeration probes (CEN) for chromosomes 4, 8 and 20, and a locus-specific identifier probe (LSI) for p53 (all Vysis, Surrey, UK) were used. Two probes were used simultaneously (20+4, p53+8). Fluorescence *in situ* hybridisation was performed according to slightly modified manufacturer's instructions. In total, 5 *μ*l of probe mixture was added to each cytodot (3.5 *μ*l hybridisation buffer, 0.5 *μ*l of each probe and 1 *μ*l of water). The sample and probe were codenatured on a 75°C hotplate for 2 min and incubated in the dark at 37°C for 30 min (CEN) or 16 h (LSI). Slides were washed for 2 min at 73°C with 0.4 × SSC/0.3% Nonidet P-40, and for 30 s at room temperature in 2 × SSC/0.1% Nonidet P-40, and allowed to air dry in the dark. To counter stain the nuclei, 10 *μ*l Dapi (Vysis, Surrey, UK) was added to each slide.

### Signal visualisation and scoring

An Olympus BX50 microscope and Powergene 4.3 software (Applied Imaging, Newcastle Upon Tyne, UK) were used to score each slide. Slides with <100 cells were excluded and an average of 223 cells per slide was scored (range 100–452). In total, approximately 32 000 cells were scored for chromosome abnormalities (223 cells per slide, two slides per brush, two brushes per patient (up to eight brushes per surgical patient), 37 patients). Nuclei were only included if they had at least one signal from each probe to avoid inclusion of results where hybridisation was inadequate. Nuclei that were smeared or overlapping were also excluded.

Centromeric enumeration probes highlight the centromere of the chromosome and were used to determine whole chromosome changes, that is, aneuploidy. A loss of a CEN signal was assumed to be a deletion of that chromosome, and more than two signals was determined an amplification. The LSI probe for p53 was a marker of that gene locus, and again a loss of a signal represented deletion, whereas a gain represented amplification.

Slides were coded before scoring, with no knowledge of the histological details of the tissue sample.

### Statistical analysis of chromosomal abnormalities

Fishers exact test was used to compare the chromosomal changes between the differing histological diagnoses, *P-*values <0.05 were deemed to represent significant differences.

The reproducibility of this technique was assessed by performing FISH with the same probes on different slides from the same gastric brush. No statistical differences in the levels of aneuploidy were noted.

### PCR determination of *H. pylori*

Antral biopsies were taken from patients at the time of endoscopy and DNA was extracted using a Stratagene DNA extraction kit (Stratagene, Cambridge, UK). A UV spectrophotometer (Beckman DU 530) was used to quantitate the DNA extracted from each biopsy and 200–500 ng was used for subsequent PCR analysis. *Helicobactor pylori* flagellin primers, designed by ourselves (forward: AAACCAATCGCTGTGAAACC, reverse: ACGG AAGGCTTTCTCTCACA) were used to generate a 94 base pair fragment of the flagellin gene. The CagA primers were synthesised according to Lage *et al*, 1995. Both the flagellin PCR and the CagA PCR were optimised internally such that single band PCR products were obtained. The PCR reaction comprised 1.5 mM MgCl_2_, 400 *μ*M dNTPs, 200–500 ng DNA, 2.5 U of *Taq* polymerase (Promega, Southampton UK) and *Taq*-specific 10 × buffer. The PCR was performed in a DNA engine (MJ research, Watertown, MA, USA) and the thermal profile was as follows: 94°C for 2 min and 30 cycles of 94°C for 30 s, 60°C for 20 s and 72°C for 20 s. PCR products were visualised post-PCR by PAGE using 5% polyacrylamide. The gels were run in Protean III electrophoresis systems (BioRad, HemelHempstead, UK) and poststained with silver.

### *In vitro* ROS study

Cells from the human cell line, AHH1 (Genetest Corporation), used in routine chromosome damage assays, were prepared in tissue culture and dosed with 0, 50, 100 *μ*M H_2_O_2_ (Sigma Aldrich) for 30 min, followed by 24 h recovery. Hydrogen peroxide was chosen as the model ROS due to its ease of administration and as it is thought to represent a diffusible form of ROS. This dose was chosen as it was subtoxic and has previously been shown to cause DNA/chromosomal damage (Rueff *et al*, 1993; Perwez Hussain *et al*, 1994; Duthie *et al*, 1997; Fenech *et al*, 1999; Umegaki and Fenech, 2000; Jenkins *et al*, 2001). Initially, the micronucleus assay was performed to determine if chromosome damage was induced following standard protocols (Fenech and Morley, 1985; Fenech *et al*, 1999). The micronuclei were subsequently stained with a kinetochore label to assess if whole chromosomes were present within them. Secondly, FISH analysis was performed to specifically look at copy numbers of chromosomes 4, 8, 20 and 17(p53), post H_2_O_2_ exposure.

## RESULTS

A total of 27 patients met the inclusion criteria and were enrolled at the Endoscopy department at Neath Port Talbot Hospital. Three additional premalignant patient samples were obtained from brushes taken at the Pathology Department in Morriston Hospital from surgical resections. Hence, the total number of patient's samples with premalignant gastric disease considered in this part of the study was 30, but four endoscopic patients were not included in final analysis because of insufficient cells/inadequate histology. Samples from 10 patients undergoing surgical resections of tumours were also studied. These surgical samples often supplied adjacent premalignant tissue as well as adenocarcinoma material. Details of age/sex of patients, site of lesion and histological diagnosis are illustrated in Table 1. All the stages of Correa's multistep pathway to cancer (normal gastric tissue, gastritis, intestinal metaplasia, dysplasia) could be demonstrated in some of the individual surgical resections. *Helicobactor pylori* infection was not identified in any late stage surgical sample as is usual in gastric tissue that has become malignant (Graham, 2000; You *et al*, 2000).

**Figure 1:**
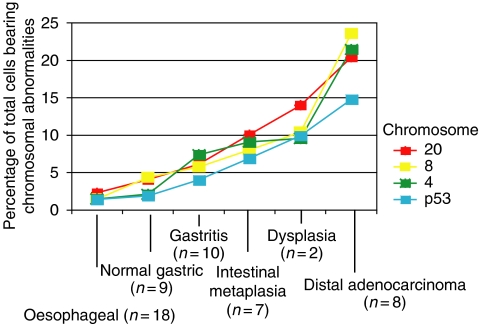
Overall aneuploidy (loss+gain) during the histological progression. All chromosome abnormalities become more prevalent during cancer progression. Interestingly, man chromosome abnormalities are more present in normal gastric tissue than normal oesophageal tissue.

**Figure 2:**
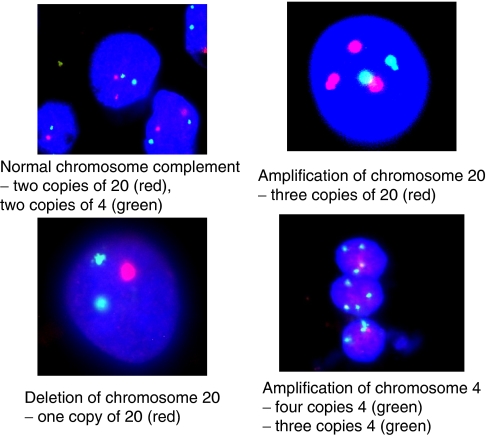
Examples of chromosomal abnormalities identified in gastric epithelial cells.

Aneuploidy was demonstrated at all stages of progression to gastric cancer, with the abundance of aneuploid cells increasing with disease severity. Also, the individual malignant gastric cells contained multiple copy number changes compared to the early premalignant tissue, in which single abnormalities tended to be seen. Interestingly, the normal gastric tissue contained higher levels of background chromosome abnormalities than the matched oesophageal samples, presumably as a result of the harsher conditions present in the stomach. The cumulative chromosome abnormality data across the histological series is shown in Figure 1. Amplification of chromosome 4, deletion of p53 and amplification of chromosome 20 were the most significant events found in the premalignant stages of gastric carcinogenesis. Figure 2 demonstrates some of the types of aneuploidy detected using FISH. Table 2 illustrates the rank order that individual amplifications and deletions appeared in the premalignant and malignant stages. As can be seen, amplification of chromosome 4, amplification of 20 and deletion/amplification of p53 are the most frequently noted events.

One of the gastric tumours studied was located at the gastro-oesophageal junction and as such had received preoperative chemotherapy. The same type of aneuploidy was seen in this tumour as in the distal cancers, but the frequency of abnormalities shown were less, possibly reflecting the effect of chemotherapy. Also, the gastric normal tissue in this patient was analysed separately and when compared to the gastric normal tissue from the other patients in this study, chromosomal abnormalities were higher. This may possibly suggest induced aneuploidy by the chemotherapy treatment, as has been shown in other studies (Acar *et al*, 2001; Kamiguchi and Tateno, 2002; Silva *et al*, 2002; Frias *et al*, 2003), but numbers are obviously too small to confirm this. Furthermore, in several cases, multiple brushes were taken from the same tumour to study intratumoural heterogeneity. There was indeed heterogeneity in the aneuploidy present in different parts of the same tumour.

### Chromosomal abnormalities in *H. pylori*-positive samples *vs H. pylori*-negative samples

**Figure 3:**
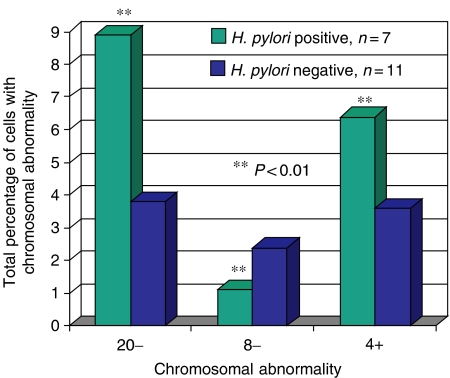
Chromosomal abnormalities separated by *H. pylori* status showing significant differences in abundance. Chromosome 20 deletion and 4 gain are more prevalent in *H. pylori*-positive samples, whereas deletion of 8 is more prevalent in *H. pylori-*negative samples.

The identification of *H. pylori* in the endoscopic cohort of patients (i.e., gastritis and IM only) was determined using PCR as well as histology in 18 patients. The use of PCR here can improve the detection rates by 10% or so (Ishmail, 2004). In all, 39% of the 18 patients (seven out of 18) attending the endoscopy clinic were *H. pylori* positive by PCR and histology. All patients with *H. pylori* infection had abnormal gastric tissue. Figure 3 illustrates the significant increases in levels of aneuploidy present in *H. pylori-*positive patients (*n*=7) as compared to *H. pylori-*negative individuals (*n*=11). CagA status was also determined within this subset, with three out of seven infections possessing CagA. The numbers of patients in each group here are too small to make firm conclusions, but increased aneuploidy was present with the CagA+ strains, notably involving chromosomes 4 and 8.

### *In vitro* analysis of chromosomal abnormalities induced by ROS

**Figure 4:**
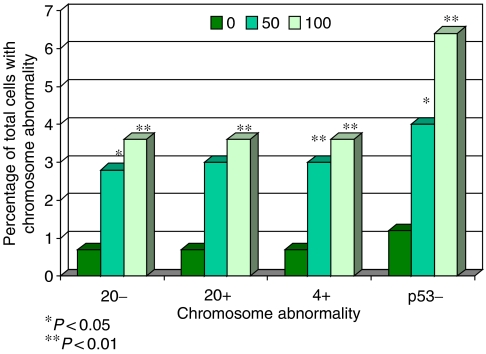
Fluorescence *in situ* hybridisation data from AHH-1 cells exposed to H_2_O_2_ for 30 min, showing similar chromosomal changes to those detected in gastric tissue *in vivo*.

The *in vitro* study using the micronucleus assay to determine the effects of ROS exposure on human cells demonstrated, as expected, that chromosome damage increased with ROS dose. Kinetochore staining confirmed that these micronuclei often contained whole chromosomes. Hence, ROS exposure lead to the production of aneuploidy. Fluorescence *in situ* hybridisation analysis further illustrated that abnormalities of chromosomes 20, 8, 4 and 17(p53) were induced by ROS exposure. Figure 4 summarises the FISH data showing the statistically significant chromosome abnormalities induced in the cell line by this particular ROS.

## DISCUSSION

We show here that interphase FISH, in conjunction with brush cytology, successfully allowed karyotype changes, in both premalignant and malignant gastric tissue, to be monitored. This approach has previously been developed to analyse aneuploidy in oesophageal tissue (Doak *et al*, 2003). If an association between aneuploidy in premalignant cells and gastric cancer risk were to be established, this approach may be suitable as a surveillance tool. The most important adaptation needed for extending the method to gastric tissue involved accounting for intrinsic cell digestion by gastric juice. Interestingly, we show here that background aneuploidy levels in histologically normal gastric tissue were elevated compared to normal oesophageal tissue. This is presumably due to the harsh environment endured by the gastric cells, hence, the suggestion is that acid or bile, key agents in gastric contents, may play a role in inducing aneuploidy in gastric tissue. Bile and acid have previously been implicated as having a role in the mechanism of upper GI cancer. Bile acids in particular have been shown to cause DNA damage (Scates *et al*, 1995, 1996) and chromosomal aberrations (D’Souza *et al*, 2003).

Gastric cancer presents late, and as such, treatment options are limited and currently survival rates are poor. Improving the outcome of cancers can often be achieved by identifying people at a high risk of developing cancer (Hussain and Harris, 1998) and by early cancer detection (Matturri *et al*, 1998). The latter has been addressed in Japan, where the incidence of gastric cancer is one of the highest worldwide, and screening programmes have been developed to diagnose lesions early, improving the 5-year survival rates to 95%. Unfortunately, only 10% of European gastric cancers are detected early (Keighley, 2003). The identification of molecular markers to determine early disease may be one approach whereby survival rates could be improved. Identifying the actual mechanisms that lead to the aneuploidy demonstrated in early gastric carcinogenesis may even help to identify therapeutic agents capable of halting the carcinogenic process at its earliest stages. The detection of chromosomal instability, even in premalignant stages, as found in this study, may help to explain the aggressive nature of this disease. Furthermore, intratumoural heterogeneity was also detected in a number of the tumours analysed. This phenomenon has been recognised in other gastrointestinal cancers, reflecting the general instability of the tumour, and the development of *de novo* genetic abnormalities in individual cells. Owonikoko *et al* (2002) demonstrated this phenomenon in Barrett's associated cancers, using a number of genetic markers, whereas Lindforss *et al* (2000) has shown similar events in cancers of the lower GI tract. If a tumour shows significant intratumoural heterogeneity, then the likelihood is that at least one of the various clones of cells present will survive chemotherapy and the cancer will continue to grow.

There were no associations between the aneuploidy detected in this study and the smoking history of patients, or sex, age or diet. However, there was a strong association with the histological stage of the gastric tissue, and aneuploidy events increased in frequency as the tissue histology approached cancer. Gastric cancer is said to be a predominantly male disease (Sipponen *et al*, 1998; Faraji and Frank, 2002; Grady, 2001), however, in the premalignant cohort studied, only 33% were male increasing to 50% in the cancer group. The locality in which the cohort was recruited has the highest percentage of female gastric cancer patients in the country (www.wcisu.nhs.uk). Also, the cancer group was almost a decade younger then that expected nationally (www.wcisu.nhs.uk), suggesting a possible local predisposition to this form of cancer.

Of particular interest in this study was the amplification of chromosome 4, which was detected with increasing frequency as premalignant gastric tissue tended towards gastric adenocarcinoma, suggesting that it may have an important link with the initiation and development of gastric cancer. Chromosome 4 amplification has been recently shown to be a significant event in the progression of Barrett's oesophagus to adenocarcinoma, especially in the early histological changes of this pathway (Croft *et al*, 2002; Doak *et al*, 2003). The level of chromosome 4 amplification in the intestinal metaplasia samples studied here (7%) was less than, but nonetheless comparable with, that seen in the metaplasia patients with Barrett's disease (12%) (Doak *et al*, 2003). Further work by our group has suggested the link between the amplification of chromosome 4 and nuclear factor-*κ*B (NF-*κ*B) (Jenkins *et al*, 2004). Nuclear factor-*κ*B resides on chromosome 4 and NF-*κ*B acts as an antiapoptotic factor, as well as influencing the proinflammatory action of IL-8 (Rayet and Gelinas, 1999). The increased copy number of NF-*κ*B in cells with a chromosome 4 amplification may allow continued cell survival in the presence of noxious gastric secretions. This aspect warrants further investigation. A recent report has illustrated gene expression changes induced by coculture of AGS cells with *H. pylori* and attempted to correlate these expression changes with known cytogenetic anormalities in gastric cancer (Myllykangas *et al*, 2004). In the paper by Myllykangas *et al*, six genes on chromosome 4 show expression changes after coculture with *H. pylori*, with three genes upregulated and three genes actually downregulated. Hence, this does not fit well with our cytogenetic data which showed only amplification of chromosome 4. However, comparing *in vitro* models of gastric cancer and *in vivo* gastric tissue itself is obviously not ideal.

Changes in chromosome 20 copy number have previously been demonstrated in gastric cancer. In this study, amplification of chromosome 20 was found frequently in premalignant tissue. Panani *et al* (1995) have shown with CGH that amplification of chromosome 20 can occur in gastric adenomas as well as gastric carcinoma. In our study, abnormalities of chromosome 20 were usually found to be more frequent than other chromosomal abnormalities, also having a higher prevalence in normal gastric tissue. This could therefore be representative of general instability of the tissue, and be a marker of aneuploidy, that is, chromosome nondisjunction, due to increased cell proliferation. As for candidate genes on chromosome 20, which could explain its amplification, it is interesting to note that the Aurora kinase A gene has been located to chromosome 20q13.2. Aurora kinase A has been found previously to be overexpressed in tumours and in cancer cell lines (Giet *et al*, 1999; Zou *et al*, 1999; Carmena and Earnshaw, 2003) and is involved in the separation of centrosomes as well as spindle assembly (Carmena and Earnshaw, 2003). Hence, amplification of chromosome 20 may reflect abnormalities of this protein kinase, whose altered function may have a role in the further development of aneuploidy, and hence genetic instability in carcinogenesis. In the paper by Myllykangas, eight genes on chromosome 20 show expression abnormalities, five genes were upregulated and three genes downregulated. Indeed, one particular locus on chromosome 20 (20pter-p11) showed the largest number of upregulated genes. These upregulations *in vitro* may be linked to the amplification of chromosome 20 *in vivo*, as they would both cause increased activity of particular genes at these loci.

P53 deletion was the other significant abnormality that emerged during this study of gastric carcinogenesis, although p53 gain was also evident in tissue of more advanced histological disease, perhaps reflecting more general chromosome 17 aneuploidy. p53 is involved in the control of cell cycle progression and apoptosis in response to DNA damage, and hence loss of its function would lead to genetic instability (Grady, 2001). This tumour suppressor gene is the most frequently altered gene in human cancer (Levine *et al*, 1991) and loss of p53 is a frequent finding in gastric cancer (Grady, 2001). In this study, loss of p53 was shown to be a frequent abnormality in premalignant tissue. Many studies have demonstrated abnormalities of p53 in gastritis (Morgan *et al*, 2003), and intestinal metaplasia (Uchino *et al*, 1993; Shiao *et al*, 1994) as well as gastric cancer. In Myllykangas's paper, 22 genes on chromosome 17 show expression changes, 15 of them upregulation. This may reflect the late stage amplification of 17p that we detected here and could involve the erbb-2 gene (17q12–21). Three of the genes in Myllykangas's paper are present at 17p13–11, a similar locus as p53 and two of three of these genes show upregulation after incubation with *H. pylori*. This disparity may be due to *in vitro* *vs in vivo* factors.

Alterations in chromosome 8 copy number were also found in premalignant and malignant gastric tissue, their prevalence increasing with the histological progression. Other studies have suggested that amplification of 8 is more frequent in advanced cancers and therefore may be a late event (Wu *et al*, 2001). Both deletions and amplifications were seen in this study in all tissue types investigated, but amplification of 8 was seen in up to 20% of cancers. Chromosome 8 houses an important oncogene, c-myc, which acts as a proliferative transcription factor. Its overexpression has been observed in many tumour types, including gastric cancers (Grady, 2001). There are some reports of abnormalities of c-myc being present in the premalignant stages of gastric cancer. Panani *et al* (1995) have shown gains of chromosome 8 in gastric adenomas. One study reports frequencies of c-myc overexpression in 36% of gastric cancers, and 15% of chronic gastritis. All these abnormalities were also associated with abnormal p53 expression (Nardone *et al*, 1999). The significant link between *H. pylori-*negative patient tissue and chromosome 8 loss has an unknown basis that might be worth pursuing. In Myllykangas's paper, eight genes on chromosome 8 show expression abnormalities, five upregulation and three downregulation, reflecting our mixed cytogenetic data.

Identification of *H. pylori* infection in this study was determined by histology and PCR. Previous work by a number of groups has supported the accuracy of PCR diagnosis of *H. pylori* infection (Clayton *et al*, 1992; Lage *et al*, 1995; Ashton-Key *et al*, 1996). The prevalence of *H. pylori* infection in this cohort of patients undergoing upper GI endoscopy was 39%, and this is comparable to the findings of a similar endoscopy-based study from the same hospital in which multiple gastric biopsies were analysed for *H. pylori* using PCR (Balaratnam *et al*, 2001). This infection rate is also comparable to another European study looking at infection rates in patients undergoing endoscopy that found 39% of gastric biopsies had PCR evidence of *H. pylori* infection (Lage *et al*, 1995). The further identification of the CagA strain by PCR was undertaken, as it is known to be associated with greater disease severity. The prevalence of the CagA strain of *H. pylori* in this study was 43%, which is lower than in some of the other studies reported but comparable to a local study in which only gastritis patients were analysed, 44% of *H. pylori* infections in that study were CagA+ (Morgan *et al*, 2003). Aneuploidy of chromosomes 4, 20 and 8 were more evident in *H. pylori-*positive tissue (Figure 3). Abnormalities of p53 have already been associated with *H. pylori*-positive infections (Murakami *et al*, 1999). In particular, in this study, chromosome 4 amplification was significantly increased in infected tissue. The link between *H. pylori* infection, an agent known to be causally linked to gastric cancer, and the amplification of chromosome 4 suggests the importance of *H. pylori* as an initiating agent in gastric carcinogenesis, and the amplification of 4 as an early genetic event. Aneuploidy of chromosomes 4, 8 and 20 was also seen at a higher frequency in those gastric biopsies infected with CagA+ *H. pylori* strains, but numbers here were too small to make firm conclusions. This supports previous data that has demonstrated that CagA+ strains were associated with greater levels of DNA aneuploidy, and p53 and c-myc expression (Nardone *et al*, 1999). Given the possible link between H. *pylori* strains and chromosome 4 amplification, it is interesting to note that CagA infection has already been associated with greater IL-8 levels (Crabtree *et al*, 1995). It is well known that NF-*κ*B controls IL-8 gene expression and hence CagA+ strains may induce IL-8 upregulation via an NF-*κ*B and chromosome 4-dependent mechanism.

*Helicobactor pylori* is known to cause inflammation and the release of ROS in gastric tissue (Bagchi *et al*, 1996; Farinati *et al*, 1998; Obst *et al*, 2000). Studies have shown that ROS and its subsequent formation of oxidative DNA damage are important factors in gastric cancer development (Correa and Shiao, 1994; Stadtlander and Waterbor, 1999). Our *in vitro* study here has demonstrated the types of aneuploidy induced by a model ROS, H_2_O_2_, in a cell line routinely used in assessing chromosome damage. Hydrogen peroxide was chosen as it has been found *in vivo*, and therefore mimics the actual type of ROS released during tissue inflammation (Fenech *et al*, 1999; Marnett, 2000). The types of chromosomal abnormalities induced by this model ROS study were similar to those found in gastric carcinogenesis, but most comparable with gastritis. This is not surprising given that gastritis is a highly inflammatory condition possessing high levels of ROS (Marnett, 2000; Morgan *et al*, 2003).

Finally, it should be reiterated that even though a considerable amount of FISH data has been accumulated, patient numbers in this study were relatively small, especially when the cohort was subdivided for analysis. As such, the observations made here should be confirmed with larger studies targeted at the key chromosomal abnormalities detected, which may represent useful molecular markers for the future. Furthermore, it would be important to repeat this study in histology sections to closely match the cytogenetic information with the histology. Amplification of chromosome 4 and its effect on NF-*κ*B and IL-8, would be important to follow up, as would the association with *H. pylori* infection. Also, aneuploidy of chromosome 20, and the possible relationship to Aurora kinase A and the development of further aneuploidy, is of particular interest.

In conclusion, chromosomal instability has been demonstrated in early gastric carcinogenesis and this may be the reason why gastric cancer is such an aggressive disease.

## Figures and Tables

**Table 1:** Patient characteristics as subdivided by histological diagnosis

	**Number**	**Mean age**	**% Male**	**% Smoking**	**% Alcohol excess**	**% Family history**	**% *H. pylori* infection**
Gastric normal	9	67	12.5	50	12.5	12.5	0
Gastritis	10	66	40	20	0	30	50
Intestinal metaplasia	4	69	50	25	50	12.5	16.6
Dysplasia (*n*=1)/cancer (*n*=9)	10	63	50	No information	No information	No information	0

**Table 2:** Fold increase in chromosomal abnormalities as disease severity worsens in gastric tissue

**Chromosomal abnormality**	**Increase in gastritis cf. normal gastric**	**Increase in IM cf. normal gastric**	**Increase in dysplasia cf. normal gastric**	**Increase in cancer cf. normal gastric**
4 gain	× 4.7	× 5.5	× 6.2	× 15.0
20 gain	× 2.9	× 7.9	× 16.4	× 20.0
P53 loss	× 2.2	× 2.9	× 4.4	× 6.0
8 loss	× 1.5	× 2.2	× 2.5	× 2.6
4 loss	× 1.4	× 2.0	× l.6	× 2.3
8 gain	× l.3	× l.7	× 2.3	× 6.5
P53 gain	× 1.3	× 5.5	× 7.0	× 13.0
20 loss	× l.2	× l.3	× 0.7	× l.8
